# Clinical Significance of Surgical Intervention to Restore Swallowing Function for Sustained Severe Dysphagia

**DOI:** 10.3390/jcm12175555

**Published:** 2023-08-26

**Authors:** Hiroaki Ito, Asuka Nagao, Suguru Maeda, Maya Nakahira, Masamitsu Hyodo

**Affiliations:** 1 Department of Otolaryngology, Kochi Medical School, Kohasu, Okou-cho, Nankoku 783-8505, Japan; hiroaki_ito@kochi-u.ac.jp (H.I.); nagaoa@kochi-u.ac.jp (A.N.); jm-smaeda@kochi-u.ac.jp (S.M.); 2 Department of Rehabilitation, Kochi Medical School Hospital, Kohasu, Okou-cho, Nankoku 783-8505, Japan; jm-nakahira-m@kochi-u.ac.jp

**Keywords:** dysphagia, surgical intervention, oral food intake, cricopharyngeal myotomy, laryngeal elevation, vocal fold medialization

## Abstract

Owing to rapid population aging, patients with dysphagia are significantly increasing in society. Dysphagia treatment is aimed at the restoration of the swallowing function and the prevention of recurrent aspiration-induced pulmonary infection. However, despite intensive rehabilitation, oral food intake remains inadequate in many patients with severe dysphagia, which results in the deterioration of patients’ quality of life and joy of living. Surgical intervention may serve as a useful therapeutic strategy to restore swallowing function in these patients. The study included 25 patients (mean, 70.4 years; male/female ratio, 20:5) with chronic dysphagia. Dysphagia was associated with cerebrovascular diseases in sixteen patients; with age-induced physiological deterioration in five patients; and with miscellaneous etiologies in four cases. Cricopharyngeal and infrahyoid myotomies were performed in all patients. Laryngeal elevation and the medialization of the paralyzed vocal fold were performed in 15 and 3 patients, respectively. The Food Intake Level Scale (FILS) and videoendoscopic examination score (VEES) were used to evaluate swallowing function. The FILS showed a restoration of oral food intake alone in 72% of patients, and 64% of patients maintained this improvement at their last follow-up visit. We observed significantly improved VEES scores postoperatively. However, patients with cognitive impairment or advanced age showed poor outcomes. In conclusion, surgical intervention may be an effective therapeutic option to restore swallowing function in cases of sustained severe dysphagia; however, surgical indications require careful consideration.

## 1. Introduction

Owing to rapid population aging, swallowing disorders secondary to stroke, neuromuscular disorders, head injury, head and neck cancer, dementia, and aging have become important medical and social concerns [1,2,3]. Patients with severe dysphagia, particularly elderly individuals, are unable to consume food orally and are at risk of life-threatening aspiration pneumonia or choking. Pneumonia is a leading cause of death in Japan [4], and most patients with pneumonia have dysphagia [5]. Therefore, treatment in patients with swallowing disorders is aimed at the restoration of oral food intake and the prevention of aspiration pneumonia. Primary treatments for dysphagia include food texture modification, oral hygiene measures, pharmacological therapy, and swallowing rehabilitation [1,3,6]. However, conservative treatments may be inadequate for patients with severe or progressive dysphagia, in whom chronic dysphagia is associated with a reduced quality of life, physical deterioration, and increased caregiver burden.

Surgical intervention is an alternative treatment for severe dysphagia [7,8,9] and includes the following procedural concepts: (i) the restoration of oral intake and (ii) the prevention of intractable aspiration pneumonia. The former procedure preserves laryngeal functions such as phonation and physiological respiration. It includes laryngeal elevation, cricopharyngeal myotomy, and vocal fold medialization [8]. In this study, we retrospectively investigated the postoperative outcomes in patients who underwent surgery to restore swallowing function with preserving laryngeal functions, and we discuss the clinical role and indications for surgery.

## 2. Materials and Methods

### 2.1. Patients

The study included 25 patients who underwent surgery to restore swallowing function at Kochi University Hospital between 2008 and 2018. All patients received conservative treatments, including food modification and swallowing rehabilitation for sustained severe dysphagia for more than several months (3–49 months, 8.4 months in average). Dysphagia was associated with stroke (cerebral infarction or hemorrhage) in 16 (including 8 cases of Wallenberg syndrome), aging in 5 (including 3 cases of disuse syndrome), and complications of long-term intubation, pharyngoesophageal diverticulum, and cricopharyngeal muscle myopathy secondary to sarcoidosis and scleroderma with anterior cervical osteophytes in 1 patient each.

### 2.2. Surgical Procedure

Details of the surgical procedures have been reported previously [7,8]; therefore, we present only a brief outline of these procedures.

#### 2.2.1. Cricopharyngeal Myotomy

Cricopharyngeal myotomy was performed in patients with impaired opening of the upper esophageal sphincter (UES). The transcervical approach was used, and the cricopharyngeal muscle was resected bilaterally in a 3 cm long, 1 cm wide size to avoid reattachment of the cut end of the muscle. The lower part of the thyropharyngeal and upper part of the esophageal muscle were included in the resection because of the indistinct boundaries between the cricopharyngeal and the thyropharyngeal or esophageal muscles [10].

#### 2.2.2. Laryngeal Elevation

The thyroid cartilage was suspended anterosuperiorly and fixed to the anterior mandible to ensure sufficient laryngeal elevation (thyromandibular approximation). Two Teflon tapes utilized to suture the uterine cervix (Sanritu Medical Instruments MFG Co., Ltd., Tokyo, Japan) were used to fix the thyroid cartilage because of its durability to maintain long-term laryngeal suspension [8]. Tracheostomy was combined with postoperative airway management for temporary pharyngolaryngeal mucosal edema. After confirming reduction in edema, the tracheostomy opening was closed within a few weeks postoperatively.

#### 2.2.3. Vocal Fold Medialization

Surgical medialization of the paralyzed vocal fold improves glottal closure and reduces the risk of aspiration in cases of unilateral vocal fold paralysis. These procedures include medialization thyroplasty [8,11], arytenoid adduction [12], and injection augmentation of the vocal folds [13]. The two former procedures were performed using a transcervical approach and the latter via a transoral approach. Patients who underwent vocal fold medialization alone to improve breathy hoarseness for unilateral vocal fold paralysis were excluded from the study.

### 2.3. Assessment of Swallowing Function

Pre- and postoperative oral intake were assessed using the Food Intake Level Scale (FILS) proposed by Fujishima [14]. Swallowing function was also evaluated using the videoendoscopic examination score (VEES) for swallowing, as proposed by Hyodo [15]. All patients were assessed preoperatively at discharge from the hospital, and during postoperative follow-up. The best and latest scale scores were recorded during follow-up. Furthermore, we focused on 16 patients with cerebrovascular disease to analyze changes.

#### 2.3.1. Food Intake Level Scale 

The FILS was introduced in 1993. This scale rates dysphagia on a scale from 1 to 10 to measure the severity of dysphagia and determine the degree of oral food consumption in patients on a daily basis (Table 1). Scores of 1–3 indicate no oral feeding, scores 4–6 indicate oral intake combined with alternative nutrition, scores 7–9 indicate oral intake alone with some restriction or consideration, and score 10 indicates normal intake. Previous studies have validated the reliability and clinical significance of the FILS tool [14,16].

#### 2.3.2. Videoendoscopic Examination Score of Swallowing 

The VEES, proposed by Hyodo, is widely used in Japan as a standard evaluation tool for oropharyngeal swallowing function that assesses both the non-swallowing and swallowing phases [15,17,18]. The VEES consists of the following items that assess swallowing function: (A) salivary pooling in the vallecula and piriform sinuses, (B) elicitability of the glottal closure reflex or cough reflex by touching the epiglottis or arytenoid with the endoscope, (C) the provocation of the swallowing reflex, and (D) pharyngeal clearance after swallowing 3 mL of dyed water (Table 2). Each item is scored from 0 to 3. The maximum total score is 12 points; the higher the score, the more severe the swallowing dysfunction. Hyodo reported that a total score of ≥9 points indicates difficulty in oral feeding. Items (A) and (D) reflect pharyngolaryngeal motor function and items (B) and (C) reflect sensory function [15]. The VEES can objectively and semi-quantitatively evaluate oropharyngeal swallowing function.

### 2.4. Statistical Analysis

The Wilcoxon signed-rank sum test was used to compare changes in the FILS and VEES scores. *p* value < 0.05 was considered statistically significant. All statistical analyses were performed using the STATA statistical software ver 15.1 (StataCorp LLC, College Station, TX, USA).

### 2.5. Ethical Statement

The protocol for this retrospective study was approved by the Institutional Review Board of the Kochi Medical School (31-138, 26 November 2019). We used an opt-out format, and informed consent was not obtained from each patient.

## 3. Results

### 3.1. Patient Demographics

Table 3 shows patient demographics. Patients’ ages at the time of the operation ranged from 43 to 87 years (mean 70.4 years). Most patients were in their 70 s (nine cases), followed by patients in their 80s (seven cases). The male/female ratio was 20:5. Postoperative follow-up ranged from 2 to 129 months (median 17 months). Table 4 shows the surgical procedures performed in this study. All patients underwent cricopharyngeal and infrahyoid myotomy for laryngeal elevation. Laryngeal elevation surgery was performed in 15 patients. Vocal fold medialization (medialization thyroplasty in one and arytenoid adduction in two patients) was performed in three patients with glottic insufficiency secondary to unilateral vocal fold paralysis. The remaining three patients underwent pharyngeal constriction surgery on the paralyzed side, a resection of anterior cervical osteophytes, and pharyngoesophageal diverticulectomy. Tracheostomy was performed in all patients who underwent laryngeal elevation surgery.

### 3.2. Evaluation of Oral Intake Ability Using the Food Intake Level Scale

**Figure 1:**
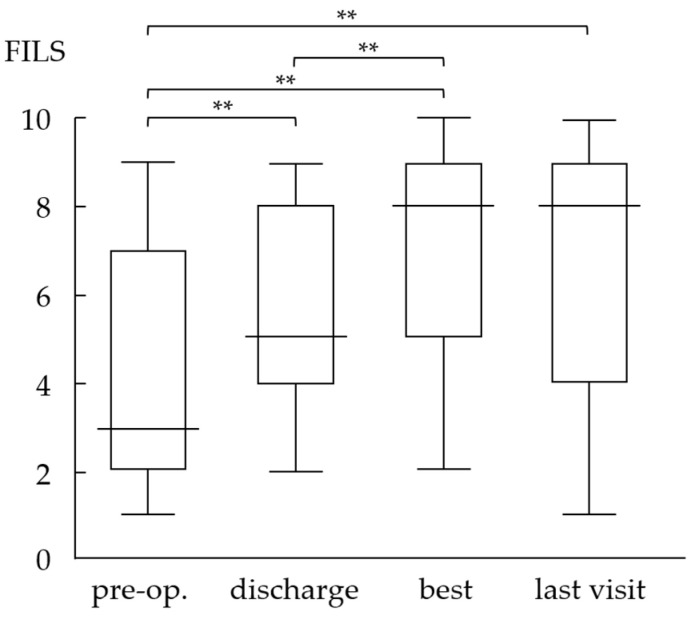
Pre- and postoperative Food Intake Level Scale scores. The FILS scores show significant improvement at discharge and during postoperative follow-up. FILS: Food Intake Level Scale, **: *p* < 0.01.

The mean FILS scores improved significantly at discharge from the hospital (5.4), the best visit (7.1), and the last visit (6.7) compared with scores at the preoperative visit (4.0) (Figure 1, *p* < 0.01). The score was significantly higher at the best visit than at hospital discharge (*p* < 0.01). Among the 25 patients included in the study, 10 (40%) were able to consume all meals orally (FILS ≥ 7) at the time of discharge; however, this figure increased to 18 (72%) at the best visit and 16 (64%) at the last visit. Preoperatively, 17 patients were dependent on tube feeding; among these, 16 had minimal oral intake (FILS scores 1–3) and one patient required combined tube feeding (FILS score 6). Postoperatively, 65% (*n* = 11) of the patients at the best visit and 59% (*n* = 10) of patients at the last visit could consume food orally without additional feeding support. Only one patient (4%) was unable to resume oral food intake.

**Figure 2:**
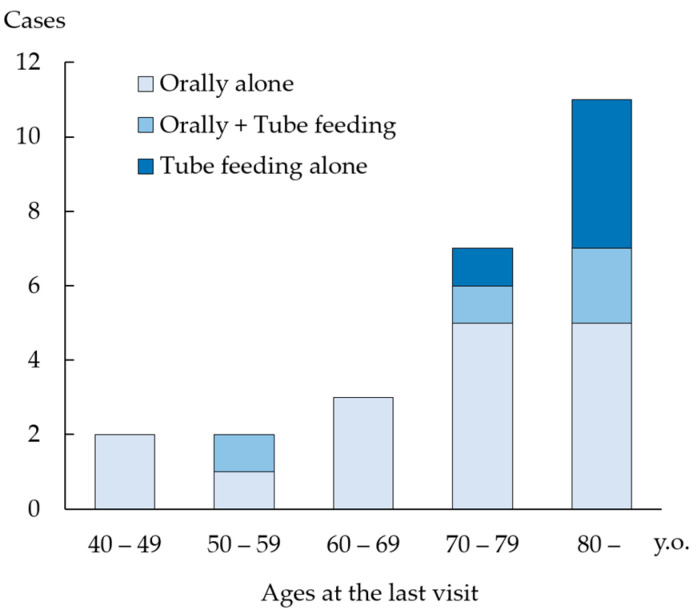
Food intake status by ages at the last visit. Among patients aged <70 years at the last visit, 86% of patients (6/7) were able to consume food orally without feeding support. However, 71% of patients (5/7) in their 70 s and only 45% of patients (5/11) in their 80 s or older at the last visit could consume food orally without feeding support.

During follow-up, swallowing ability gradually deteriorated, so that four patients (16%) who had once resumed oral intake were unable to take food orally at the last visit. These patients could not complete sufficient postoperative swallowing rehabilitation, owing to physical or cognitive impairment or restriction of oral intake, considering the risk of aspiration based on nursing home policies. These patients were aged >74 years. With regard to age, 86% of patients (6/7) aged <70 years at the last visit were independent of tube feeding (Figure 2), whereas 71% of patients (5/7) in their 70 s and only 45% of patients (5/11) aged ≥80 years at the last visit could maintain independent oral food intake.

### 3.3. Videoendoscopic Examination of Swallowing Score

**Figure 3:**
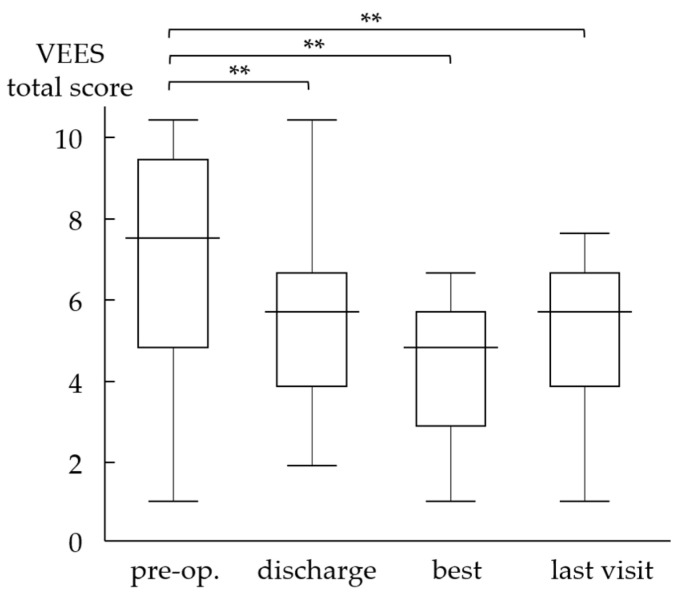
Pre- and postoperative videoendoscopic examination scores. The VEES sores show significant improvement at discharge and during postoperative follow-up, although slight worsening is observed at the last visit compared with scores at the best visit. VEES: Videoendoscopic examination score, **: *p* < 0.01.

**Figure 4:**
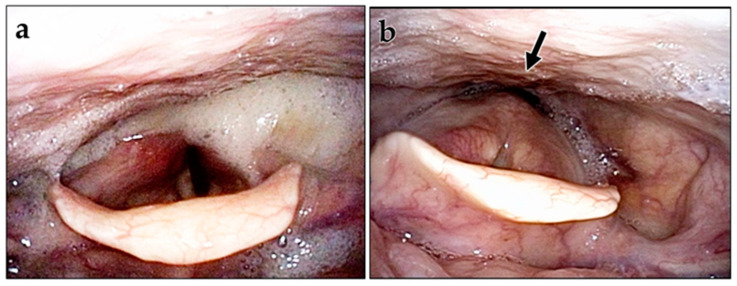
Pre- and postoperative endoscopic findings of the pharyngolarynx of a patient with left brainstem infarction. (**a**) Preoperative image showing much pooling of saliva in the piriform sinuses. (**b**) Postoperative image showing a small amount of saliva retained in the piriform sinuses with an open esophageal inlet (arrow).

The VEES showed significant improvement with a total score of 6.0 at discharge, 4.7 at the best, and 5.4 at the last visit compared with 7.7 preoperatively (Figure 3, *p* < 0.01). We also observed a significant difference between scores at discharge and at the best time point (*p* < 0.01). Figure 4 shows the pre- and postoperative endoscopic images after cricopharyngeal myotomy and laryngeal elevation in a patient with left brainstem infarction. Among these patients, nine exhibited a worsening of their scores at the last visit compared with their best visit scores; however, no patient had a VEES ≥ 9, which renders patients ineligible for oral intake. Notably, six of the nine patients maintained an FILS score of ≥8, whereas the other three patients showed a decrease in the FILS score to 4 or 5. These patients were elderly or cognitively impaired, with age and Mini-Mental State Examination Scores as follows: 72 years and 17/30, 78 years and 22/30, and 84 years and 27/29.

### 3.4. Investigation of Patients with Stroke

We investigated 16 patients with stroke, which is known to cause dysphagia (often chronic dysphagia). The mean FILS score was 3.1 preoperatively and significantly improved across the following time points during follow-up: 5.3 at discharge, 7.2 at the best, and 7.1 at the last visit. We compared postoperative FILS scores between the groups with less and severely impaired sensory function of the pharyngolarynx to investigate the association between the VEES and surgical efficacy and oral food intake. In the less-impaired sensory group (*n* = 7), the total score of (B) elicitability of glottal closure/cough reflex and (C) the provocation of the swallowing reflex was ≤3, the postoperative mean FILS score at the best visit was 8.1; whereas in the severely impaired sensory group (*n* = 9), the total score of (B) and (C) was ≥4, it was 6.7. Although the difference was statistically nonsignificant (*p* = 0.17), patients with less sensory impairment tended to show better outcomes.

### 3.5. Postoperative Complications and Outcomes

Four patients (16%) developed local infection around the Teflon tape used for laryngeal elevation. In each patient, the Teflon tape was removed unilaterally 26–159 days postoperatively, and the infection subsided. No patient developed a worsening of swallowing function after tape removal. The removal of cervical vertebral osteophytes was associated with unsatisfactory outcomes in a patient who underwent cricopharyngeal surgery; therefore, this patient underwent additional surgery with laryngeal elevation, which led to the successful restoration of oral intake. The tracheostomy wound was closed postoperatively in 8 of 15 patients (53%) who previously underwent the procedure or underwent tracheostomy concomitant with the current surgery. Six patients died during postoperative follow-up: four patients died of causes unassociated with dysphagia and two died of aspiration pneumonia.

## 4. Discussion

In this study, we retrospectively reviewed the postoperative course in patients with severe chronic dysphagia, who underwent surgery to restore swallowing function. We observed improvement in the FILS and VEES scores in many patients, even in those with an inability to consume oral food; however, some patients showed unsatisfactory results. We discuss the role and indications of surgical intervention for the treatment of dysphagia.

### 4.1. Role of Surgical Intervention

Oral food intake is a primary physiological function in humans and symbolizes individuals’ quality of life and the joy of living. Therefore, the restoration of oral intake is the most important goal of treatment in patients with dysphagia. Surgical interventions such as aspiration prevention surgeries, which include total laryngectomy [19,20], tracheoesophageal diversion [21,22], laryngotracheal separation [23,24], and laryngeal closure [25,26], are performed in patients with dysphagia. These procedures can successfully prevent the aspiration of the bolus and restore oral feeding [27,28]. They also significantly reduce burdens on the patients, caregivers, and families [27,28,29]. However, patients who undergo these surgeries lose vocal function and require permanent tracheostomy. The loss of voice negatively affects patients’ quality of life and results in a considerable decline in their overall well-being and may lead to estrangement owing to the failure of effective communication. They also inevitably suffer from an impaired sense of smell and taste, susceptibility to tracheal and bronchial infections, and the inability to blow. Surgical interventions to restore oral intake with preserving laryngeal functions were developed in the 1950s–1970s. They primarily aim to compensate for pharyngolaryngeal swallowing motor dysfunction by strengthening laryngeal closure, increasing the pharyngeal driving force of food, and assisting UES opening with the preservation of physiological functions of the larynx [8]. These procedures score over aspiration prevention surgery in selected patients with severe dysphagia. However, surgical interventions to restore swallowing function has received little attention. This is because the indications for surgery are not clear, the surgical technique and postoperative management are rather complex, and alternative feeding, such as gastrostomy, has been considered to complete the treatment.

### 4.2. Purpose of Surgical Procedures

Cricopharyngeal myotomy, which dilates the contracted UES and improves the passage of food through the upper esophageal opening is the most well-known procedure performed for dysphagia [7,8]. This approach was first reported by Kaplan [30], and many authors have subsequently reported its efficacy [9,31]. In this study, all patients underwent cricopharyngeal myotomy, and this operation should be considered a fundamental surgical procedure in such cases. Recently, endoscopic cricopharyngeal myotomy has been demonstrated to be a safe and efficacious procedure with favorable outcomes for the treatment of cricopharyngeal dysfunction [32,33]. Although all our patients underwent open surgery for combined operations of infrahyoid myotomy and/or laryngeal elevation, the endoscopic approach has the advantage of being less invasive. Laryngeal elevation is the second most frequently performed procedure in patients with dysphagia. Shifting the larynx anterosuperiorly results in the backward bending of the epiglottis to close the laryngeal opening, and the esophageal inlet widens anteriorly to open it [7,8,34]. Inadequate glottal closure during swallowing predisposes to aspiration in patients with unilateral vocal fold paralysis. Additionally, vocal fold paralysis is accompanied by breathy hoarseness and difficulty expectorating the aspirated material from the trachea. Surgical medialization of the paralyzed vocal fold strengthens glottal closure during swallowing and phonation and can effectively overcome these concerns [7,8,13]. Pharyngeal paralysis in patients with vagal nerve paralysis causes inadequate pharyngeal constriction, which is necessary to propel food into the esophagus; therefore, pharyngeal residue leads to post-swallow aspiration. In such cases, unilateral pharyngeal constriction surgery increases the pharyngeal swallowing pressure and reduces the pharyngeal residue. Infrahyoid muscles, including the sternohyoid, sternothyroid, and omohyoid draw the larynx downward. Sections of these muscles aid laryngeal elevation. These procedures were selected and combined in our patients based on functional impairment observed in each case.

### 4.3. Surgical Outcomes

Compared with preoperative scores, postoperative FILS scores showed significant improvement across all time points during postoperative follow-up for the restoration of swallowing function in this study. At the last visit, 64% of patients were able to consume food orally without additional feeding support, although we observed a slight deterioration compared with the condition at the best time point. Among patients who were completely unable to consume food orally preoperatively, 59% were able to consume food orally at the last visit, which underscores the favorable effects of surgical intervention. The favorable postsurgical outcomes were more prominent in patients with stroke accompanied by stable non-progressive dysphagia. The improvement of oral food intake ability led to a considerable improvement of the patients’ quality of life. VEES (a tool for objective assessment of swallowing function) also improved significantly postoperatively. However, the FILS and VEES scores showed less improvement in patients aged >75 years and in those with cognitive impairment, which is attributable to inadequate postoperative swallowing rehabilitation in these patients. These findings indicate that in addition to an appropriate surgical procedure, postoperative swallowing rehabilitation is mandatory to achieve a satisfactory restoration of the swallowing function.

### 4.4. Surgical Indications

Surgical interventions to improve swallowing function aim to compensate for or augment impaired motor function during the pharyngeal stage [8]. Therefore, it is necessary to comprehensively understand the pathophysiology of dysphagia before surgery. Both VE and videofluorographic examinations are essential to evaluate laryngeal elevation, UES opening, pharyngeal constriction, and the elicitability of the swallowing reflex. Manometric examination is also used to evaluate pharyngoesophageal movements, particularly the pattern of transmission of swallowing pressure and UES opening [35,36].

However, pharyngolaryngeal dysfunction cannot be corrected to its original state. Therefore, postoperative swallowing rehabilitation is mandatory to improve oral intake ability. However, a fairly well-maintained consciousness level, cognitive function, and physical motor function are essential to perform such rehabilitation. In this study, patients with impaired cognitive function had poorer outcomes. Age is an important prognostic factor. In the present study, the rate of improvement was lower with increasing age, particularly in patients aged >80 years; we treated seven patients in their 80 s, and surgical outcomes were unfavorable in all seven. Highly aged patients often experience physical and psychological deterioration, poor adherence to rehabilitation, and a low willingness to consume oral food. Therefore, we recommend an age criterion of 75 years (preferably age ≤ 70 years) for surgical intervention to restore swallowing function.

In this study, improvement in FILS scores tended to be greater in patients with better glottal closure and swallowing reflexes. Optimal pharyngolaryngeal sensory function is essential to receive mechanical, thermal, and chemical stimuli to detect the presence of the food bolus to trigger the swallowing reflex. This reflex is also important to prevent aspiration and to facilitate the expectoration of aspirated foods or liquids [37,38]. Surgical intervention does not improve sensory function; therefore, it should be maintained when considering indications for surgery to improve swallowing function. A thorough evaluation of the sensory function of the pharyngolarynx using VE or other modalities is important [39,40,41]. Patients’ living and nursing environments after hospital discharge are important factors; a lack of support necessary for oral intake and daily living results in the worsening of swallowing ability. Careful long-term follow-up in cooperation with the family, caregivers, and physicians is essential after discharge.

Based on this study, we propose the following indications for surgery to improve swallowing function:

(1) Unsatisfactory improvement in swallowing function despite professional conservative treatments such as rehabilitation.
(2) Stable and non-progressive primary disease.
(3) Dysphagia that is confirmed as primarily a disorder of the pharyngeal swallowing phase.
(4) Patient age ≤ 70 years.
(5) Fairly well-maintained physical and cognitive functions.
(6) Fairly well-maintained pharyngolaryngeal sensation.
(7) Willingness for oral food intake.
(8) Availability of the necessary support at the patient’s home or in a nursing home.

### 4.5. Limitations

The following are the limitations of this study: (a) We followed strict surgical indications; therefore, the study included a relatively small number of patients. However, we objectively assessed swallowing function chronologically and observed the successful restoration of oral intake ability even in patients with severe dysphagia. (b) The retrospective study design is a drawback, and patient characteristics were fairly diverse. Further prospective studies are warranted to strengthen the effectiveness of intervention and to establish the criteria for surgical intervention in patients with severe chronic dysphagia refractory to conservative treatment. (c) Many physicians are unfamiliar with this surgical approach for the treatment of patients with dysphagia. It is necessary to improve awareness regarding the therapeutic effectiveness of surgical interventions in patients with long-lasting severe dysphagia. Also, the organization of a surgical training course or the creation of video materials would contribute to their promotion.

## 5. Conclusions

This study highlights that surgical intervention improved both the long-term FILS and VEES scores postoperatively in patients with severe chronic dysphagia. The surgical approach restores oral food intake and also the joy of living, which reiterates its usefulness as an effective treatment option. However, patients of advanced age and those with cognitive impairment and poor support tend to show unfavorable surgical outcomes. Surgical intervention is associated with favorable outcomes; however, indications for surgery require careful consideration.

## Figures and Tables

**Table 1:** Food Intake Level Scale (FILS).

**No Oral Intake**		
	Level 1:	No swallowing training ^#1^ is performed except for oral care.
	Level 2:	Swallowing training not using food is performed.
	Level 3:	Swallowing training using a small quantity of food is performed.
**Oral Intake and Alternative Nutrition**		
	Level 4:	Easy-to-swallow food ^#2^ less than the quantity of a meal (enjoyment level) is ingested orally.
	Level 5:	Easy-to-swallow food is orally ingested in one to two meals, but alternative nutrition is also given.
	Level 6:	The patient is supported primarily by ingestion of easy-to-swallow food in three meals, but alternative nutrition ^#3^ is used as a complement.
**Oral Intake Alone**		
	Level 7:	Easy-to-swallow food is orally ingested in three meals. No alternative nutrition is given.
	Level 8:	The patient eats three meals by excluding food that is particularly difficult to swallow ^#4^.
	Level 9:	There is no dietary restriction, and the patient ingests three meals orally, but medical considerations ^#5^ are given.
	Level 10:	There is no dietary restriction, and the patient ingests three meals orally (normal).

^#1^ Swallowing training: Training conducted by an expert, well-instructed caregiver, or the patient himself/herself to improve the swallowing function. ^#2^ Easy-to-swallow food: Food that is prepared so that it is easy to swallow even without mastication, for example, meat and vegetables are gelatinized or homogenized in a mixer. ^#3^ Alternative nutrition: Non-oral nutrition such as tube feeding and drip infusion. ^#4^ Food that is particularly difficult to eat: Dry and brittle food, hard food, water, and so on. ^#5^ Medical considerations: Guidance, tests, examinations, and so on, for symptoms suggestive of swallowing disorders such as choking and the feeling of food remaining in the pharynx.

**Table 2:** Videoendoscopic examination score for swallowing (VEES).

**(A) Salivary pooling in the vallecula and piriform sinuses**	
Score 1	No pooling.
2	Mild pooling.
3	Moderate pooling to fill the piriform sinus, but penetrate into the larynx.
4	Severe pooling to overflow into the larynx during inspiration.
**(B) Elicitability of glottal closure reflex or cough reflex**	
Score 1	Easily elicited by a soft touch on the epiglottis.
2	Elicited by touches on the epiglottis but weak.
3	Sometimes not elicited by touches on the epiglottis, elicited by touches on the arytenoid.
4	Very poorly elicited.
**(C) Provocation of swallowing reflex**	
Score 1	Only slight pharyngeal inflow of dyed water can be observed before white-out ^#1^.
2	Dyed water can be observed to flow to the vallecula.
3	Dyed water can be observed to flow into the piriform sinus.
4	Swallowing reflex hardly occurs for a while after dyed water flows into the piriform sinus.
**(D) Pharyngeal clearance after 3 mL of dyed water swallowed**	
Score 1	No residue.
2	Mild residue, can be cleared by a few additional swallowing.
3	Moderate residue, cannot be cleared by a few additional swallowing.
4	Severe residue with considerable aspiration into the larynx.

^#1^ White-out is defined as a brief interruption of endoscopic image by pharyngeal closure.

**Table 3:** Demographics of the patients.

	(*n* = 25)
Age (y.o.)	43–87 (mean: 70.4)
Male/Female	20:5
Postoperative follow-up (months)	2–129 (median: 17)
Cause of dysphagia:	
Cerebrovascular disorder	16
Aging	5
Long-term intubation	1
Pharyngo-esophageal diverticulum	1
Sarcoidosis cricopharyngeal myopathy	1
Scleroderma	1
FILS (pre.)	1–9 (mean: 4.0)
VEES (pre.)	1–11 (mean: 7.7)

FILS: Food Intake Level scale; VEES: videoendoscopic examination score.

**Table 4:** Surgical procedures for the patients.

Surgical Procedure						
Cricopharyngeal myotomy	○	○	○	○	○	○
Laryngeal elevation		○	○			
Vocal fold medialization			○			
Pharyngeal constriction				○		
Cervical osteophyte resection					○	
Pharyngo-esophageal diverticulectomy						○
Infrahyoid myotomy	○	○	○	○	○	○
*n*	5	12	3	2	2	1

## Data Availability

The raw data will not be shared due to ethical approval constraints. However, the datasets generated and/or analyzed during the current study are available from the corresponding author on reasonable request.
